# Complete Genome Sequences of seven cluster DE1 phages isolated using *Gordonia terrae*

**DOI:** 10.17912/micropub.biology.001909

**Published:** 2025-11-20

**Authors:** Azzam Almutairi, Gabriella Marsala, Natalie Mershon, Sarah Ball

**Affiliations:** 1 Department of Biochemistry, The Ohio State University, Columbus, Ohio, United States; 2 Department of Psychology, The Ohio State University, Columbus, Ohio, United States; 3 Center for Life Sciences Education, The Ohio State University, Columbus, Ohio, United States; 4 Department of Molecular Genetics, The Ohio State University, Columbus, Ohio, United States

## Abstract

The phages Barsten, Kamashten, Kwobi, LilHam, SchottB, StorminNorm and Thing3 were isolated from soil samples collected around Columbus, Ohio, United States, using the host *Gordonia terrae* 3612. The seven genomes range in size between 55,593bp and 59,501bp, and contain an average of 85 protein-coding genes. Based on gene content similarity, all seven phages are assigned to actinobacteriophage cluster DE1.

**Figure 1. Genomic characteristics of isolated phages:**
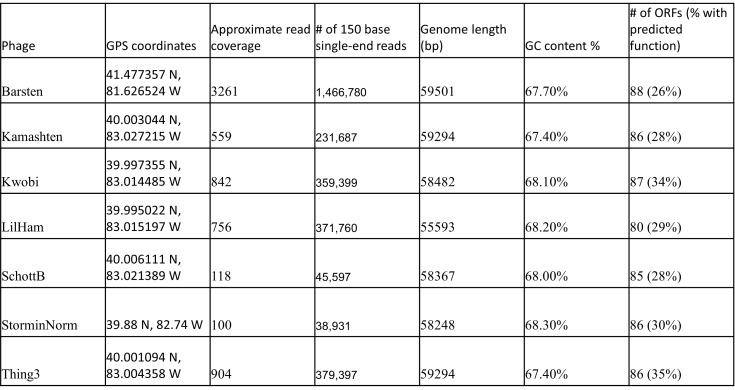
The sequencing and genome information for seven DE1 phages isolated using *Gordonia terrae* 3612, including read coverage and number of 150 base singe-end reads, genome length, GC content, and number of predicted open reading frames.

## Description

*Gordonia spp.* are gram-positive bacteria belonging to the phylum Actinobacteria. Some members of this genus may have environmental and biotechnical uses due to their ability to break down natural polymers and pollutants, while others are opportunistic pathogens (Arenskötter et al. 2004). To better understand the diversity of bacteriophages capable of infecting *Gordonia spp* ., seven phages were isolated and purified from soil samples using the host bacterium *Gordonia terrae* 3612.

All seven of the phages were isolated from soil samples collected in Columbus, Ohio, United States, following standard protocols (Zorawik et al. 2024)). Isolation was achieved by shaking the soil sample with peptone-yeast extract-calcium (PYCa) liquid media for 1 hour, then centrifuging the samples for 10 minutes at 2,000 x g. The supernatant was filtered through a 0.22 μm filter and plated in PYCa top agar with *Gordonia terrae* 3612. The plates were incubated at 30°C for 7 days. The phages were purified through three rounds of plaque streaking. Following incubation, StorminNorm and Thing3 produced slightly turbid plaques, while the other phages produced clear plaques. Plaque sizes ranged from 2.0 to 3.0mm. All the phages exhibit siphovirus morphology.

Phage DNA was extracted from a lysate using a Promega Wizard DNA Clean-Up kit. The DNA was prepared for sequencing with the NEB Ultra II Library Kit and sequenced with an Illumina MiSeq 1000 sequencer (v3 reagents). The raw reads were assembled by Newbler v2.9 (Miller et al., 2010) with default settings and Consed v29 (Gordon and Green, 2013) was used to check accuracy, coverage, and genomic termini (Figure 1). The result was genomes with circularly permuted ends. All seven phages were assigned to cluster DE1 based on gene content similarity (GCS) above 35% to phages within the Actinobacteriophage Database (Russell & Hatfull, 2017; Pope et al. 2017). The genomes ranged from 59,501 bp to 55,593 bp in length, with an average GC content of 67.9% (Figure 1).

The genomes were automatically annotated in DNAMaster v5.23.6 (Pope and Jacobs-Sera, 2018) using Glimmer v3.0 (Delcher et al. 2007) and GeneMark v2.5 (Borodovsky, 2005). The annotations were refined through BLAST searches against the NCBI nonredundant and actinobacteriophage databases (Altschul et al., 1990), and HHpred searches against these four databases: PDB_mmCIF70, SCOPe70, Pfam-A, and NCBI_Conserved_Domains (CD) (Söding et al., 2005). Phamerator (Cresawn et al., 2011), using the Actino_draft database and TMHMM were also employed, with default settings. No tRNAs were detected with Aragorn v1.2.38 (Laslett and Canback, 2004) or tRNAscan-SE v2.0 (Lowe and Eddy, 1997). The percentage of genes assigned a putative function ranged from 26%-35% (Figure 1). The absence of an integrase gene supports the prediction that all seven phages are lytic and cannot produce lysogens, a feature shared by members of the DE1 cluster. LilHam and SchottB each contained one orpham (gene with no known homologs), while the other phages contained none. All predicted protein-coding genes are transcribed in the same direction, with no significant gaps between open reading frames. All seven genomes display the typical order of genes found in cluster DE1, with structural genes on the left arm, followed by various tail proteins and the lysis cassette, which includes both lysin A and lysin B. The nucleotide similarity of the right arm of the genomes shows less conservation between the phages, however they all contain genes involved in DNA metabolism.

Nucleotide sequence accession numbers

Barsten is available at GenBank Accession No. MT498035 and Sequence Read Archive (SRA) No. MT498035

Kamashten is available at GenBank Accession No. PV915807 and Sequence Read Archive (SRA) No. PV915807

Kwobi is available at GenBank Accession No. PV915880 and Sequence Read Archive (SRA) No. PV915880

LilHam is available at GenBank Accession No. PV915824 and Sequence Read Archive (SRA) No. PV915824

SchottB is available at GenBank Accession No. OQ995426 and Sequence Read Archive (SRA) No. OQ995426

StorminNorm is available at GenBank Accession No. OQ995426 and Sequence Read Archive (SRA) No. OQ995426

Thing3 is available at GenBank Accession No. OQ995426 and Sequence Read Archive (SRA) No. OQ995426
